# Editorial: Recent Progress and Perspectives in Neurosteroid Research

**DOI:** 10.3389/fendo.2022.951990

**Published:** 2022-07-27

**Authors:** Hubert Vaudry, Takayoshi Ubuka, Kiran K. Soma, Kazuyoshi Tsutsui

**Affiliations:** ^1^Université de Rouen Normandie, Mont-Saint-Aignan, France; ^2^International Cancer Laboratory, Tokyo, Japan; ^3^University of British Columbia, Vancouver, BC, Canada; ^4^Hiroshima University, Hiroshima, Japan

**Keywords:** neurosteroids, neurohormones, neuromodulators, neurotransmitters, neurotrophic factors

The term "Neurosteroids" designates pregnenolone-derived bioactive compounds that are synthesized or catalyzed by neural cells and act locally on the central or peripheral nervous systems. At the molecular level, the actions of neurosteroids are mainly mediated through membrane receptors i.e. via their cognate receptors and/or via allosteric modulation of other receptors. At the cellular level, neurosteroids act as neurohormones, neuromodulators, neurotransmitters and/or neurotrophic factors. At the organismal level, neurosteroids regulate several physiological processes including arousal, sleep, learning, social and sexual behaviors. Thus, neurosteroids are now considered an important class of intercellular/intracellular signaling molecules in the nervous system, in very much the same as neurotransmitters, neuropeptides and growth factors. Not surprisingly, neurosteroids appear to be implicated in a number of pathophysiological conditions such as pain, neurodegenerative diseases, autism, stress, anxiety, depression, etc. Therefore, drugs targeting neurosteroid biosynthetic enzymes or neurosteroid receptors have strong potential for the development of novel therapeutic approaches.

This Research Topic compiles a series of review and original articles that provide a broad view of the current knowledge on the biosynthesis, functional roles and pathophysiological implications of neurosteroids, and highlights new concepts in this field.

We want to dedicate this Research Topic to the memory of our colleague and dear friend Kazuyoshi Tsutsui, an undisputed leader in neurosteroid research who, very sadly, passed away on September 16, 2021.

Progesterone and allopregnanolone are two neuroactive steroids that act primarily through membrane receptors (1). Progesterone can activate five metabotropic membrane receptors belonging to the progestin and adipoQ receptor (PAQR) family that are distinct from the GPCR family (2). Allopregnanolone and its 3β-methylated synthetic analog ganaxolone act as positive allosteric modulators of GABA_A_ receptors (Pinna). The review by Thomas and Pang summarizes the current knowledge on the neuroprotective actions of allopregnanolone and ganaxolone.

Concurrently, Pinna describes the long journey between the discovery of allopregnanolone in the adrenal cortex (3) and the characterization of its anxiolytic and antidepressant properties (4, 5). The recent validation of allopregnanolone-based treatment of postpartum depression (6) opens new avenues for the development of other neurosteroid-derived drugs in neuropsychiatry.

Apolipoprotein A1 regulatory protein-1 (ARP-1), a member of the steroid receptor superfamily whose ligand is unknown (orphan receptor), regulates transcriptional activity of numerous genes including apolipoprotein-encoding genes (7). Using a yeast one-hybrid screening approach, Honda and Harada identified ARP-1 as a transcription factor that binds to a *cis*-element, aro-AII, responsible for the brain-specific expression of the mouse aromatase gene.

The recent years have seen substantial progress in the understanding of the bidirectional interactions between the hypothalamo-pituitary-adrenal (HPA) and the hypothalamo-pituitary-gonadal (HPG) axes (8). Hamidovic et al. have conducted a meta-analysis of data spanning over half a century of research on plasma cortisol levels in healthy women during the follicular *vs.* luteal phases of the menstrual cycle. Their study reveals that circulating cortisol levels are higher in the follicular phase. One possible explanation relies on the changes of GABA_A_ receptor-modulating neurosteroids, including allopregnanolone, during the menstrual cycle (9).

GABAergic anesthetic agents such as isoflurane can exert adverse neuroendocrine effects, notably during the neonatal period (10). Li et al. have investigated the effects of sevoflurane on testosterone (T) and its derivative 17β-estradiol (E2) levels in 5-day old rats. They found that sevoflurane causes an increase in T levels in male rats only and an increase in E2 levels in both male and female rats. These data indicate that the adverse effects of general anesthesia at the beginning of the lifespan might be ascribed, at least in part, to a GABA_A_ receptor-mediated increase of plasma sex steroids.

Biosynthesis of estrogens is catalyzed by the enzyme cytochrome P450 aromatase, also called estrogen synthase, that converts T into E2 (11). A recent study has revealed that administration of aromatase inhibitors may impair cognitive functions (12). In this context, Alia-Klein et al. have taken advantage of the availability of positron emission tomography (PET) aromatase radiotracers to correlate cognitive performance with aromatase levels in the human amygdala and thalamus.

During the perinatal period, neuroestrogens play a pivotal role in the sexual differentiation of the brain (13, 14). The review by Tsukahara and Morishita discusses the role of neuroestrogens of testicular origin on two sexually dimorphic brain regions, the preoptic area (POA) and the bed nucleus of the stria terminalis (BNST). Surprisingly, during the peripubertal period, testicular androgens, without aromatization, also contribute to sexual differentiation of the POA and BNST.

There is now strong evidence that estrogens synthesized within the CNS exert a neuroprotective action (15). Reciprocally, traumatic brain injury (TBI) can affect both estrogen biosynthesis and estrogen inactivation in the central nervous system (CNS) (16). Indeed, TBI can modulate various estrogen metabolizing enzymes including aromatase, steroid sulfatase, estrogen sulfotransferase and 17β-hydroxysteroid dehydrogenases.

Neuroactive steroids, like steroid hormones, can act through intracellular receptors (genomic actions) and/or plasma membrane receptors (non-genomic actions) (17). Neurosteroids are not only synthesized in the CNS but also in the peripheral nervous system (PNS) (18). Colciago et al. provide a comprehensive review of the various aspects of neurosteroid actions in the PNS through intracellular receptors, metabotropic receptors (*i.e.* G protein-coupled receptors) and ionotropic receptors (mainly GABA_A_ receptors).

Various neurosteroids can negatively or positively modulate GABA_A_ receptor activity and can thus act as proconvulsant or anticonvulsant agents (19). In their systematic review, Miziak et al. summarize the diverse effects of endogenous and exogenous neurosteroids on seizure activity in animal models and epileptic patients.

Astrocytes express both cytochrome P450scc and 3β-hydroxysteroid dehydrogenase, the two enzymes that are required for the biosynthesis of progesterone (20). Although estradiol initiates the luteinizing hormone (LH) surge that triggers ovulation and reproduction, estrogens do not act directly on gonadotropin-releasing hormone (GnRH) neurons (21). Sinchak et al. review the evidence that neuroprogesterone synthesized in hypothalamic astrocytes is involved in the estradiol-induced LH surge.

Fish, which exhibit intense aromatase activity in their CNS (22), represent attractive models to investigate the effect of neuroactive steroids on behavior. Silva et al. reviews the contribution of a weakly electric teleost fish, *Gymnotus omarorum*, to the understanding of the neuroendocrine mechanisms underlying non-breeding aggressive behavior. The data strongly support the involvement of brain-born estrogens in year-long territorial behavior.

In birds, the biosynthesis of various neurosteroids is higher in the pineal gland than in any other brain region. In particular, 7α-hydroxypregnenolone (23) and allopregnanolone (24) are actively produced in the chicken pineal gland. Haraguchi and Tsutsui review the physiological roles played, respectively, by 7α-hydroxypregnenolone and allopregnanolone in the control of locomotor activity and in Purkinje cell survival during development.

In human as in other vertebrates, sex steroids affect multiple neural and behavioral functions (25). Since the menopause transition is associated with a drop in estrogen levels, He et al. have performed functional MRI scan on premenopausal and perimenopausal women to investigate spontaneous brain activity. The results reveal altered brain functions in brain regions implicated in cognition and working memory in perimenopausal women.

The post-menopausal syndrome includes various neuropsychological disorders including depression, anxiety and dementia (26). In order to investigate the role of estrogens in these disorders, Renczès et al. have compared the effects of surgical (ovariectomy) and pharmacological (aromatase inhibitor) treatment on anxiety-like behavior and memory.

Neuroactive estrogenic and androgenic neurosteroids enhance hippocampal memory tasks (27). In their mini-review, Tozzi et al. recapitulate the evidence supporting the involvement of E2 and T in the induction of long-term potentiation (LTP) and long-term depression (LTD), and on dendritic spine formation in different brain areas. The data indicate that, while estrogens induce LTP and androgens induce LTD, both neurosteroids enhance dendritic spine formation.

Do transient changes in hormonal state during the menstrual cycle affect human behaviors? To answer this question, two types of experimental designs can be set up: within-subject designs where the same women are tested during different phases of their cycle or between-subject designs where two groups of women are tested at different cycle phases. Here, Diekhof et al. have performed a between-subject study to explore the effect of hormonal changes during the late follicular phase and the mid luteal phase on avoidance learning capacity, and they have concurrently conducted a meta-analysis of previously reported within-subject studies. Both approaches concur to demonstrate a decline in avoidance learning during the follicular phase compared to the luteal phase.

In conclusion, the contributions gathered in this Research Topic give an overview on recent advances in our understanding of the physiological roles and potential therapeutic implications of neurosteroids. They also highlight the challenges that remain to be addressed for the next decade. It is our hope that the readers will enjoy reading these articles, and that this Research Topic will become a major set of references for all researchers involved in this rapidly expanding field.

## Author Contributions

All authors listed have made a substantial, direct and intellectual contribution to the work, and approved it for publication.

## Conflict of Interest

The authors declare that the research was conducted in the absence of any commercial or financial relationships that could be construed as a potential conflict of interest.

## Publisher’s Note

All claims expressed in this article are solely those of the authors and do not necessarily represent those of their affiliated organizations, or those of the publisher, the editors and the reviewers. Any product that may be evaluated in this article, or claim that may be made by its manufacturer, is not guaranteed or endorsed by the publisher.
